# Chinese Taoist Cognitive Therapy for Symptoms of Depression and Anxiety in Adults in China: A Systematic Review and Meta-Analysis

**DOI:** 10.3389/fpsyg.2020.00769

**Published:** 2020-04-23

**Authors:** Yudan Ding, Li Wang, Jindong Chen, Jingping Zhao, Wenbin Guo

**Affiliations:** ^1^Department of Psychiatry, The Second Xiangya Hospital of Central South University, Changsha, China; ^2^National Clinical Research Center on Mental Disorders, Changsha, China; ^3^Department of Psychiatry, The Second People's Hospital of Yibin, Yibin, China

**Keywords:** Taoism, Chinese Taoist cognitive therapy, culture, clinical/non-clinical depression, chronic physical diseases

## Abstract

**Background:** Chinese Taoist cognitive therapy (CTCT), a culture-oriented psychological therapy for Chinese mental well-being, has been proposed for decades. However, the evidence for its effects is unclear. This study aimed to systematically assess the effect of this therapy on symptoms of depression and anxiety in Chinese adults.

**Methods:** Relevant studies were searched from major electronic databases through November 2018 without language limits. Several search terms used include “anxiety” OR “depression” AND “Taoism” OR “Daoism” OR “Chinese Taoist Cognitive Therapy.” A total of 11 clinical trials focusing on CTCT were included in this meta-analysis. Random-effects meta-analytical models were conducted. Heterogeneity and publication bias were also explored.

**Results:** Eight clinical trials for 580 subjects were included. The majority of these studies explored samples with depressive symptoms. Overall, CTCT significantly reduced depressive symptoms with a small positive effect (SMD = 0.16, 95% CI: −0.36–0.68). Medium-to-large effect sizes were observed across individuals with clinical or non-clinical depression and chronic physical diseases (SMD = 0.70, 95%CI: 0.27–1.13 and SMD = 0.72, 95%CI: 0.09–1.35, respectively). However, the effectiveness for anxiety symptoms remains debatable.

**Conclusions:** Our findings hold promise that CTCT can help reduce depressive symptoms in Chinese adults, including patients with chronic physical diseases and clinical or non-clinical depression. Our findings may be generalized to Chinese communities in other countries.

## Introduction

### Psychotherapy for Depressive and Anxiety Symptoms

Depressive and anxiety disorders are two common types of psychiatric disorders that increasingly contribute to the global disease burden (Guo et al., 2016; Pols et al., 2018). The prevalence of anxiety disorders ranges from 2.4 to 18.2% across countries (Maguire et al., 2018). Results of the first-ever nationwide study of China's mental health showed that the most common mental disorders in China between 2013 and 2015 were anxiety disorders with a weighted prevalence of 5.0%. Weighted 12-months prevalence of depressive disorders and major depressive disorder were 3.6 and 2.1%, respectively (Huang, 2019). The findings of this survey also suggested that the prevalences of most mental disorders such as depression and anxiety increased, thereby posing considerable challenges to health-care professionals. In clinical environments, these two disorders that demonstrate pervasive mood disturbances, cognitive symptoms, and some vegetative symptoms (Guo et al., 2015) are consistently comorbid with each other and with other psychiatric or physical diseases such as substance abuse (Wu et al., 2015), diabetes mellitus type 2 (DM2), and coronary heart disease (CHD) (Pols et al., 2018). These complications negatively affect individuals and the social lives of patients and their families. Psychotropic drugs and psychological and physical treatments, alone or in a combination, can generally be used to treat depressive and anxiety disorders. Psychological intervention may be a more appropriate approach for individuals who have experienced some sub-clinical symptoms of depression and anxiety given that they may not need to take psychotropic drugs. This group of people, although diagnosed as having no mental disorders, continues to suffer from undesirable socio-occupational functioning and is at risk for full-blown depressive or anxiety disorders (Firth et al., 2019). Therefore, it is important to provide preventive treatment for these people to improve life quality and ameliorate trajectory and outcome of diseases.

### Chinese Taoist Cognitive Therapy

Cross-cultural variations are obvious not only in the presentation of different symptoms but also in psychotherapy theories and approaches (Jacob and Kuruvilla, 2012). Scholars have suggested that many psychological questions and methods are not culture-free (de Oliveira and Nisbett, 2017). Whether and to what extent a kind of psychotherapy is efficacious depends on its compatibility with individual thinking patterns, cultural values and beliefs (Benish et al., 2011). For example, cognitive behavioral therapy (CBT), originated from western cultures, is one of the most widely studied psychotherapies and increasingly adopted in Chinese societies. A meta-analysis has revealed that CBT with appropriate cultural adaptations has better therapeutic efficacy for Chinese participants than unadapted CBT (Ng and Wong, 2018). It is still unclear what types of cultural adaptations of CBT are suitable for Chinese people and how they influence the effects of treatment. The heterogeneity of regions, patients, and beliefs mandates the need to conduct appropriate culture-specific psychological interventions, by which therapists can understand patients correctly and help solve their problems. Chinese Taoist cognitive therapy (CTCT) was founded by Chinese psychiatrist Desen Yang and colleagues in 1998 (Zhang and Yang, 1998). It is a systematic culturally-grounded indigenous psychotherapy based on Taoism (or Daoism), an Eastern philosophical tradition that has a profound influence on Chinese people (de Figueiredo and Gostoli, 2013). CTCT blends features of cognitive therapy with Chinese traditional Taoism, and it attempts to help people find and modify maladaptive preoccupations and cognitive distortions by guiding them to understand main articles of Taoism and encouraging them to consider a Taoist way or behave in such a manner. Taoism, together with Confucianism and Chinese Buddhism, has a profound influence on Chinese culture and societal virtues. The three religions persist in their own independent views and attempt to pursue harmonization and convergence (Meister and Copan, 2010). Generally, Chinese people value integrity, respect and public interests rather individualism. Taoism emphasizes action without intention (called “Wu Wei”) instead of the rigid social order in Confucianism. It advocates naturalness, compassion, frugality, and humility. According to Inglehart et al. (Inglehart, 1997; Inglehart and Baker, 2000), traditional values are sustained despite economic developments and political changes. In addition, the disparities between different traditional values of a given community are much smaller than differences across nations. Even in China today, when Chinese cultures interweave with Western cultures and traditional values collide with modern values, traditional values are more pronounced in China compared with western countries (Maercker et al., 2015) and Taoism still have impact on behaviors and mental health of most Chinese people regardless of age and different experience (Yip, 2004; Wenzel-Teuber and Strait, 2012). Specifically, the older generation may be more apt to Taoistic coping style; while the younger generation, who considers Chinese culture as the priority culture, may shift between different value systems (Yip, 2004). Although there are some common factors shared between Western and non-Western psychological therapies, such as respecting patients unconditionally, demolishing dysfunctional patterns and providing new perspectives, cultural sensitivity, and cultural competency should not be neglected, especially the later one, which has become an explicit goal in psychotherapy (Jacob and Kuruvilla, 2012). Therapists should be aware of attributions of patients and their cultural organization and worldviews. Compared with other forms of psychotherapy originated from Western societies, Taoism emphasizes context and relationships, and upholds a compromise approach to contradiction (de Oliveira and Nisbett, 2017). To this extent, CTCT is suitable for Chinese adults.

**Figure 1:**
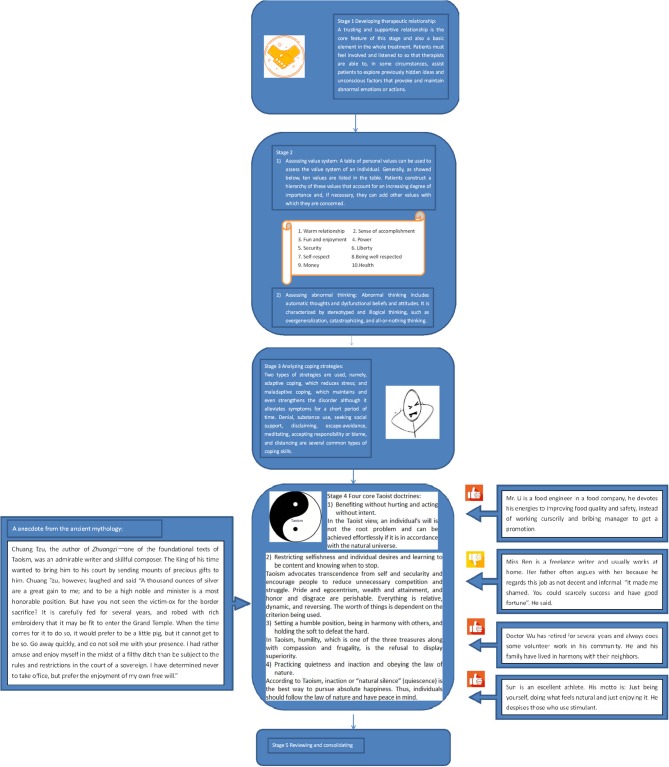
Five stages of Chinese Taoist cognitive therapy.

It has five stages; the process is sometimes called the ABCDE approach. The initials refer to actual stress, belief system, conflict and coping style, doctrine direction, and effect evaluation (See Figure 1).

Stage 1: Patients are helped to identify and record problems or life events that cause distress (e.g., bereavement and chronic disease), situations or objects that induce anxiety, and accompanying thoughts, behaviors, and emotions noted in the first stage. The Life Event Scale, a 48-item adjusted Chinese version of the Holmes and Rahe Stress Scale, is usually used to measure and give a rough picture of individual stress.

Stage 2: Value systems and ways of thinking of patients are revealed in the second stage. Value systems, the proscriptive and prescriptive belief, determine behaviors and attitudes preferred by individuals (Maercker et al., 2015). Specifically, personal values that are entangled with moral and religious tenets provide an internal reference for the behavioral patterns of individuals (what they do and in what order) by comparatively ranking values. Abnormal thinking can be exposed by observations or special interviewing skills such as laddering in the interview as well.

Stage 3: In the third stage, patients' conflicts are analyzed and coping strategies are identified. When circumstances are out of tune with personal values, inner conflicts emerge. To attempt to master, minimize or tolerate conflicts, different people adopt different coping skills which are in relation to personality and social environment. Under therapist guidance, patients are aware of their coping styles and whether they are appropriate.

Stage 4: This stage is the core of the CTCT. Four classical Taoist doctrines of mental health summarized by the initiators are introduced to patients as a new coping skill in a simple and interesting manner by giving some examples related to daily life or historical stories (Creel, 1982). The first doctrine is benefiting without hurting and acting without intent. It is also called “wu-wei” and is the leading Taoism concept linked to water through its dynamic and yielding nature in ancient Taoist texts. In the Taoist view, the universe changes harmoniously and constantly according to its own way. Individual wills can be achieved effortlessly provided that the wills are in accordance with the natural universe. However, if someone cannot recognize that the world is under a constant state of flux and achieves his or her goals in a manner that is not in tune with the natural universe, it may introduce an unintended consequence and potentially harmful interference. The second doctrine is restricting selfishness and individual desires and being content and knowing when to stop chasing fame and wealth. Taoism advocates transcendence from self and secularity and encourages people to reduce unnecessary competition and struggle. Pride and egocentrism, wealth and attainment, and honor, and disgrace are regarded as transient phenomena. Everything is relative, dynamic, and reversing. The worth of things is dependent on the criteria being used. The third doctrine is setting a humble position, being in harmony with others, and holding the soft to defeat the hard. In Taoism, humility, which is one of the three treasures along with compassion and frugality, is defined as a refusal to display superiority. The fourth doctrine is practicing quietness and inaction and obeying the law of nature. According to Taoism, inaction or “natural silence” (quiescence) is the best way to pursuit absolute happiness; thus, individuals should follow the Law of Nature and have real peace in mind. These four doctrines are arranged in a rhythmic manner in Chinese, akin to a poem; thus, they can be remembered easily. Patients are required to (1) write the doctrines in their notebook; (2) combine memorizing of the doctrines with meditation and coordinate deep rhythmic breathing; (3) record a diary (homework) documenting their thoughts or questions about the new coping strategy compared with the old, problematic ones when practiced in daily life. Then, the therapist comments on the diary and answers the questions, aiming to monitor the therapy. Dysfunctional beliefs and assumptions, if any, are also addressed in this stage. By integration with other commonly used cognitive techniques such as neutralizing and distraction, these Taoist doctrines are capable of cognitive restructuring.

Stage 5: Finally, patients are encouraged to summarize the main content of the treatment after learning and practicing for a certain period. They may undergo changes and improvements in their function. With the help of some scales, such as Hamilton Rating Scale for Anxiety (HAM-A) and Hamilton Rating Scale for Depression (HAM-D) (Wang et al., 1993), therapists can evaluate the initial effects of this treatment, and then make plans for the following treatment.

Usually, classical CTCT includes five 60–90 min interviews and stages 1, 2, and 3 are recommended to complete in the first two interviews. Doctrine direction is the essence of the treatment; thus, two or more interviews are needed to focus on stage 4. Patients attend therapy 1 or 2 times a week on an individual or group basis. All the therapists should attend specific training about CTCT and the use of outcome measures before clinical practice. Moreover, frequent supervision of each stage by a detailed rating form is important to ensure therapist competence.

CTCT is mainly considered an alternative choice for the treatment of people with mental disorders and sub-clinical depression and anxiety in China. Its history is not long, and it remains in development. To date, a small number of studies have investigated the effectiveness of this therapy for the treatment of depressive symptoms and anxiety (Zhang et al., 2000; Zhang et al.,^2011^; Qin et al., 2003; Zhou et al., 2003, 2007, 2011; Huang and Li, 2005; Wang and Liu, 2005; Wang and Xu, 2005; Yang et al., 2005; Mao and Xiong, 2007; Wang et al., 2007; Li et al., 2008, 2014, 2018; Yu et al., 2008; Li and Chen, 2009; Liu, 2009; Zhang and Zhai, 2011; Zhang, 2013; Liu and Yu, 2015; Huang et al., 2017a,b). However, the reported outcomes were inconsistent and no systematic review has been conducted to examine the reliability of those studies and synthesize the effects of CTCT. It remains unclear whether different treatment modalities (group or individual) and different treatment sessions mediate the effects. Therefore, the object of this study is to assess the effects of CTCT compared with waitlist/inactive conditions, other psychological approaches, or pharmacological therapy on symptoms of depression and anxiety in all clinical and non-clinical adults using a meta-analytic approach.

## Methods

### Study Selection

Electronic databases, including Pubmed, Embase, and Cochrane Library, were searched for all relevant articles through to November 2018. The following search terms were used: “anxiety” OR “depression” AND “Taoism” OR “Daoism” OR “Chinese Taoist Cognitive Therapy.” In order to broaden our search, the related-articles function was used, and the reference lists of identified studies were inspected. Similarly, we searched Chinese biomedical databases including Wanfang Database, China National Knowledge Infrastructure (CNKI), and Chinese Biology Medicine Database (CBM-disc).

All included studies were required to (1) be a clinical trial; (2) use CTCT; (3) recruit adults (≥18 years old) with clinical and non-clinical symptoms of depression and anxiety; and (4) use clinically validated psychometric rating scale to score clinical response. When the same groups of subjects were reported in several studies, the most complete or recent one was used. Two authors (YD and LW) independently inspected all identified literature. If any inconsistencies were found, the reviewers discussed and consulted a senior author (WG) to reach a consensus.

### Data Extraction and Quality Assessment

Two authors (YD and LW) independently extracted and summarized the following data: intervention details (treatment types, treatment modality, and duration of the intervention), participant characteristics (number of participants, age, and sex), and outcome measures (Tables 1, 2). Studies were assessed for the level of evidence reported using the Psychological Outcome Study Methodology Rating Form, which consists of 22 items (Ost, 2008). A score in the range 0–44 was allocated to each study based on these indices. Some studies, however, might not be suitable for all 22 criteria due to their design, such as comparing the CTCT with a waiting list group; here, the maximum score possible was not 44. Thus, we recalculated the total score of each study to a percentage of their possible maximum score. Likewise, if debates occurred about the score of a study, a consensus decision was adopted.

**Table 1:** Demographic and clinical information of subjects in the meta-analysis.

**Study**	**Experimental group**				**Control group**				**Outcome measure**	**Treatment type**		**ES**
	**N1**	**Mean age (years)**	**Age range (years)**	**Sex (M/F)**	**N2**	**Mean age (years)**	**Age range (years)**	**Sex (M/F)**		**Control group**	**Experimental group**	
(Zhang et al., 2011)	30	NA	NA	16/14	30	NA	NA	15/15	SCL-90	Wait list	Taoist cognitive therapy	0.37
(Wang and Xu, 2005)	31	65	43-76	14/17	31	64	36–78	16/15	HAMD	Pharmacological therapy (Fluoxetine 20 mg/day)	Taoist cognitive therapy + Fluoxetine	0.27
(Liu and Yu, 2015)^a^	30	NA	NA	15/15	30	NA	NA	15/15	HAMD	Pharmacological therapy (Paroxetine 20 mg/day)	Taoist cognitive therapy + Paroxetine	19.98
(Zhou et al., 2011)	39	67.1 ± 7.5	≥60	18/21	38	66.5 ± 8.4	≥60	20/18	HAMD	Pharmacological therapy (Fluoxetine 20 mg/day)	Taoist cognitive therapy + Fluoxetine	0.55
(Mao and Xiong, 2007)	26	56.2 ± 2.3	50–62	18/8	23	55.4 ± 3.4	49–63	15/8	HAMD	Pharmacological therapy (Fluoxetine)	Taoist cognitive therapy + Fluoxetine	0.33
(Yang et al., 2005)	35	64.5 ± 7.9	60–75	20/15	33	63.5 ± 8.5	60–72	19/14	HAMD	Pharmacological therapy (Mianserin 30–45 mg/day)	Taoist cognitive therapy + Mianserin	1.00
(Li and Chen, 2009)	23	35.1 ± 12	NA	15/8	18	34.2 ± 11	NA	7/11	SCL-90	Other psychological therapy (Hypnotherapy)	Taoist cognitive therapy	−0.24
	20	36.1 ± 10	NA	8/12						Other psychological therapy (Hypnotherapy)	Taoist cognitive therapy + Hypnotherapy	−1.09
(Zhang et al., 2000)	49	36.3 ± 10.1	NA	29/20	48	33.6 ± 10.8	NA	23/25	SCL-90	Pharmacological therapy	Taoist cognitive therapy	−0.23
	46	34.6 ± 13	NA	28/18						Pharmacological therapy	Taoist cognitive therapy + Pharmacological therapy	−1.10
(Huang et al., 2017a)	75	60 ± 5	NA	46/29	75	61 ± 6	NA	45/30	HAMD, HAMA	Wait list	Taoist cognitive therapy	4.52
(Huang et al., 2017b)	50	65 ± 9	NA	31/19	50	66 ± 11	NA	32/18	HAMD, HAMA	Wait list	Taoist cognitive therapy	3.90
(Li et al., 2018)^b^	28	NA	NA	NA	32	NA	NA	NA	HAMD	Wait list	Taoist cognitive therapy	1.79

ES, effect size; F, Female; HAMA, Hamilton Rating Scale for Anxiety; HAMD, Hamilton Rating Scale for Depression; M, Male; m, month; N, Number; NA, not available; SCL-90, Symptomchecklist90.

a , The mean age of all the included participants was 65 years, ranging from 50–82 years, and detailed information of each group was not specified;

b *, 34 men and 26 women were included in this study, and the mean age of all the participants was 54.3 ± 13.7 years, ranging from 34–89. Detailed information of each group is not available*.

**Table 2:** Characteristics of studies included in the meta-analysis.

**Study**	**Treatment modality**	**Study quality rating**	**Number of sessions**	**Follow-up (months)**
(Zhang et al., 2000)	G	25 (0.595)	4w	0
(Wang and Xu, 2005)	G	22 (0.5)	6w	12
(Liu and Yu, 2015)	NA	23 (0.523)	4w	0
(Zhou et al., 2011)	G & I	24 (0.545)	8w	6
(Mao and Xiong, 2007)	NA	25 (0.595)	4w	2
(Yang et al., 2005)	G & I	25 (0.595)	8w	6
(Li and Chen, 2009)	G & I	26 (0.591)	4w	6
(Zhang et al., 2000)	G & I	26 (0.591)	4w	6
(Huang et al., 2017a)	G	25 (0.595)	12m	0
(Huang et al., 2017b)	G	26 (0.619)	6m	0
(Li et al., 2018)	NA	24 (0.571)	6w	0

*G, intervention administered in a group; I, intervention administered individually; m, months; NA, not available; w, weeks*.

### Statistical Analysis

All data were analyzed in accordance with the Preferred Reporting Items for Systematic reviews and Meta-Analyses guidelines (PRISMA) using STATA SE version 12.0. Given that the outcomes were continuous data [means and standard deviations (SD)] from different symptom rating scales, it is inappropriate to combine data directly. Thus, the random-effect Standardized mean difference (SMD) and 95% confident intervals (CIs) were calculated to compare differences of overall effect size between experimental and control groups (DerSimonian and Laird, 1986). SMD is regarded as the difference between two means divided by the SDs of two groups: $\frac{\mu 1-\mu 2}{s}$, where μ_1_ is the post-treatment score of experimental group, μ_2_ is the post-treatment score of control group, and s, the pooled standard deviation, is defined as $\frac{\sqrt{(n1}-1)\times SD_{1}^{2}+(n2-1)\times SD_{2}^{2}}{n1+n2-2}$, where *n*_1_ and *n*_2_ are the sample size of experimental group and control group, respectively. SD_1_ and SD_2_ are the standard deviations of experimental group and control group, respectively. Statistical heterogeneity was assessed using the chi-square test and quantified using *I*^2^ with a significant set at *p* < 0.1 (Lau et al., 1997). According to Higgins (Higgins et al., 2003), *I*^2^ <25% indicates low heterogeneity, whereas *I*^2^ more than 75% means high heterogeneity between studies. Eegg's test and funnel plot were used to determine potential publication bias (*p* < 0.05).

### Subgroup Analyses

We also conducted exploratory subgroup analyses on the between-group data to examine how effects differed when (1) in clinical anxiety disorders, MDD and non-clinical depression, and chronic physical diseases with clinical; (2) adding CTCT as an add-on treatment on the base of pharmacological therapies; (3) comparing short-term CTCT treatment (<8 weeks) to long-term treatment (≥8 weeks); (4) comparing the short-term (<6 months) efficacy of CTCT to long-term (≥6 months) efficacy.

## Results

(Figure S1) showed the flowchart of study selection based on PRISMA. An initial search identified 179 articles, of which 96 duplicates were removed, thereby resulting in 83 studies. After screening the titles and abstracts of these studies, 21 were considered eligible for full-text assessment. We reviewed these 21 studies in their entirety and excluded 10 due to their incomplete data. The remaining 11 studies were selected in the final meta-analysis. No further studies were identified by reference list screening.

### Characteristics of Included Studies

Meta-analysis was conducted on 482 subjects in the treatment groups and 408 subjects in the control groups. (Tables 1, 2) show the characteristics of the 11 recruited studies and participants. All these studies were quasi-randomized controlled trials (quasi-RCTs). Four treatment comparisons were conducted: CTCT vs. other psychological therapy, CTCT plus other psychological therapy vs. other psychological therapy alone, CTCT plus pharmacological therapy vs. pharmacological therapy alone, and CTCT vs. wait list. In the CTCT plus pharmacological therapy group vs. the pharmacological therapy group, treatment courses of the two groups were the same (both: 4 weeks). When CTCT was completed, the pharmacotherapy still continued. Two studies focused on anxiety disorders, and five conducted therapy in patients with MDD applying the Chinese Classification of Mental Disorder, Third Edition as the diagnostic instrument. Participants with symptoms of depression or anxiety in seven studies suffered from chronic conditions including stroke, DM2, and CHD. In eight studies, the HAM-D was used to measure symptoms. In particular, two of these studies employed both HAM-D and HAM-A. The remaining three studies used the Symptom Checklist-90 (SCL-90). HAM-D and HAM-A are two psychological questionnaires widely used by clinicians to quantify the severity of depression and anxiety in practice as well as for research purpose (Hamilton, 1960; Maier et al., 1988). SCL-90 is a relatively brief self-report psychometric instruments, rating a broad range of psychological and psychopathological problems (Derogatis and Savitz, 2000). The Chinese versions of these four rating scales and the Life Event Scale used in stage 1 have been studied for decades and the validity and reliability were examined (Tang, 1984a,b; Wang, 1984; Zhu, 1985; Zhang and Yang, 1988). In the majority of these studies, group treatment formats were provided. Treatment sessions and follow-up duration were varied across studies (Table 2). Study quality of included studies is low. The mean quality is 24.64 and the overall scores of each individual study range from 22 (0.5) to 26 (0.619). There are some possibilities for the low study quality. Three included studies failed to provide clear description of samples (e.g., demographics and inclusion/exclusion criteria). The outcomes of those three studies which used SCL-90 to measure symptomatic severity were not specific to symptom clusters. Furthermore, blind assessors were unavailable in these studies; therapist competence was not check or the authors did not specify it; and the number of therapists was unclear. Only one study specified the assessor training and checked inter-rater reliability, and only two studies mentioned therapist training and experience. More than half of these studies failed to report the methods used to implement the random allocation and allocation concealment. Tracing and follow-up were performed by 6 studies and only one of them accomplished 1-year follow-up.

### Main Outcomes and Subgroup Analyses

**Figure 2:**
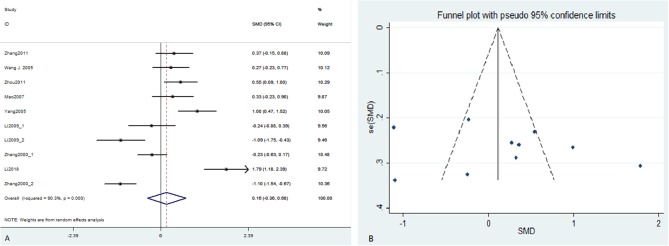
Forest plot **(A)** and funnel plot **(B)** of the meta-analysis. CI, confidence interval; SMD, standardized mean difference.

(Figure S2) showed the preliminary results of post-treatment between-group analyses of 11 studies (Supplementary Material). We excluded three studies which have remarkable influence (outliers) on the results (Liu and Yu, 2015; Huang et al., 2017a,b) according to the sensitivity analysis (Figure S3). Figure 2 and Table 3 showed the final results of 8 studies (10 datasets) when the outliers were excluded. The pooled effect size was small, but still statistically significant (SMD = 0.16, 95% CI: −0.36–0.68). The heterogeneity remained significant across those data (*I*^2^ = 90.3%). Publication bias was not detected (*p* = 0.517). In particular, CTCT plus pharmacological therapy decreased the severity of depressive symptoms more than pharmacological therapy alone (353 subjects, SMD = 0.37, 95% CI = −0.05–0.78). Heterogeneity was high (*I*^2^ = 72.9%) and publication bias was detected by the Eegg's test (*p* = 0.004). By contrast, the pooled effect size on chronic physical diseases with clinical or non-clinical symptoms of depression and anxiety was relatively large at SMD = 0.72 (95%CI: 0.09–1.35, 248 subjects). Heterogeneity was high (*I*^2^ = 82.6%) and no indication of publication bias was found using the Eegg's test (*p* = 0.400).

**Table 3:** Between-group effect sizes of included studies and subgroup analyses.

**Study**		**SMD**	**95%CI**	**Weight of included study**
Zhang et al. (2011)		0.37	−0.15, 0.88	10.09
Wang and Xu (2005)		0.27	−0.23, 0.77	10.12
Zhou et al. (2011)		0.55	0.09, 1.00	10.29
Mao and Xiong (2007)		0.33	−0.23, 0.90	9.87
Yang et al. (2005)		1.00	0.47, 1.52	10.05
Li and Chen (2009)		−0.24	−0.88, 0.39	9.56
		−1.09	−1.75, −0.43	9.46
Zhang et al. (2000)		−0.23	−0.63, 0.17	10.48
		−1.10	−1.54, −0.67	10.36
Li et al. (2018)		1.79	1.18, 2.39	9.72
Overall (*I^2^* = 90.3%, *χ^2^* = 92.94, *p* <0.0001)		0.16	−0.36, 0.68	100.00
**Subgroup Analyses**				
	**Studies/Individuals, no**.	**SMD**	**95%CI**	***I**^**2**^***, %**
CTCT + PMT vs. PMT alone	5/353	0.37	−0.05, 0.78	72.9% (*χ^2^* = 14.79, *p* = 0.005)
Anxiety disorders	2(4 datasets)/204	−0.66	−1.17,−0.15	74.3% (*χ^2^* = 11.65, *p* = 0.009)
Clinical/non-clinical depression	6/376	0.70	0.27, 1.13	75.2% (*χ^2^* = 20.13, *p* = 0.001)
chronic physical diseases	4/248	0.72	0.09, 1.35	82.6% (*χ^2^* = 17.25, *p* = 0.001)
short-term treatment (<8 weeks)	6 (8 datasets)/435	0.01	−0.60, 0.61	90.6% (*χ^2^* = 74.80, *p* < 0.0001)
long-term treatment (≥8 weeks)	2/145	0.75	0.31, 1.19	38.2% (*χ^2^* = 1.62, *p* = 0.203)
short-term (<6 months) efficacy	3/169	0.949	0.125, 1.774	84.4% (*χ^2^* = 12.86, *p* = 0.002)
long-term (≥6 months) efficacy	5 (7 datasets)/411	0.954	0.502, 1.406	80.3% (*χ^2^* = 30.48, *p* < 0.0001)

*CI, confidence interval; CTCT, Chinese Taoist cognitive therapy; PMT, pharmacological treatment; SMD, standardized mean difference*.

The pooled effect size on anxiety disorders was SMD = −0.66 (95%CI: −1.17 to −0.15, 204 subjects), thereby indicating that CTCT may not be more effective than pharmacological therapy or other psychological therapy for individuals with anxiety symptoms. By contrast, the pooled between-group effect size was SMD = 0.70 (95%CI: 0.27–1.13, 376 subjects) across subjects with clinical or non-clinical depression treated with CTCT compared with “non-active” control conditions, indicating a moderate-to-large significant effect of CTCT on depressive symptoms. The heterogeneity of the two subgroups was high (*I*^2^ = 74.3% and 75.2%, respectively) and publication bias was found in clinical or non-clinical depression group (*p* = 0.041). There was no evidence of publication bias in the anxiety group (*p* = 0.830).

As regards treatment approach, the pooled effect size of short-term treatment (<8 weeks) was very small (435 subjects, SMD = 0.01, 95% CI = −0.60–0.61) and the heterogeneity was high (*I*^2^ = 90.6%). Publication bias was not detected for this subgroup (*p* = 0.468). By contrast, significant reductions in symptoms were observed from those treated with long-term CTCT (≥8 weeks) (145 subjects, SMD = 0.75, 95% CI = 0.31–1.19) and the heterogeneity was relatively low (*I*^2^ = 38.2%) although only two studies were included in this group.

The results of efficacy analysis showed that the effect size of short-term efficacy (<6 months) and long-term efficacy (≥6 months) of CTCT on symptoms of depression and anxiety was similar (169 and 411 subjects, SMD = 0.949 and 0.954, 95% CI = 0.125–1.774, and 0.502–1.406, respectively). The heterogeneity was high in the two groups (*I*^2^ = 84.4 and 80.3%, respectively). Publication bias was not detected using the Egger's test (*p* = 0.348 and 0.540, respectively). The funnel plots of all the subgroup analyses were showed in the (Figures S4A–H).

## Discussion

To date, this systematic meta-analysis is the first to examine the efficacy of CTCT for depression and anxiety. We selected 11 quasi-RCTs studies that compared CTCT with other treatment. The results of our study indicate that, overall, CTCT is likely an efficacious add-on treatment to pharmacological therapy and more effective than inactive conditions. However, considering the high heterogeneity and unsatisfactory quality of included studies, the power of this meta-analysis was weakened. Therefore, the results should be interpreted with caution.

To explore CTCT clearly and comprehensively, we objectively stratified data and conducted several sub-group analyses. At first, the most significant result of our subgroup analyses was the positive effect of CTCT for depressive or anxiety symptoms in the context of chronic physical diseases. Many studies have demonstrated that depression and chronic diseases, such as DM2, stroke, and CHD, often co-occur and have a bi-directionally negative association (Golden et al., 2008; Pan et al., 2010). The risk of developing diabetes or macrovascular diseases is high in depressed individuals, whereas there is great risk of having comorbid depression in patients with DM2 and/or CHD (Anderson et al., 2001; Rudisch and Nemeroff, 2003; Mezuk et al., 2008; Roy and Lloyd, 2012; Gemeay et al., 2015). Furthermore, depression is responsible for lower quality of life, higher health economic burden, and reduced medication adherence and disease-related self-care activities (Gehi et al., 2005; Gonzalez et al., 2007; Nau et al., 2007; Rutledge et al., 2009; Katon et al., 2010; Chew et al., 2015; Mishra et al., 2015; Zhang et al., 2015, 2016; Mut-Vitcu et al., 2016). Evidence has underlined the need for early diagnosis and intervention of depression in patients with chronic physical diseases to improve the health outcomes of these people. This situation represents a great challenge, especially to China, which has the largest population of adult diabetics worldwide (IDF, 2017). Some meta-analyses have shown that CBT is useful to improve glycemic control and psychological conditions of patients with diabetes (Chapman et al., 2015; Uchendu and Blake, 2017). At present, apart from CBT, our results suggest that CTCT may be considered an alternative and promising intervention for Chinese patients with chronic physical diseases who suffer depressive symptoms, though the effect of selection bias cannot be eliminated due to seven of eleven included studies focusing on samples with chronic diseases to explore whether CTCT had positive effect on patients with chronic diseases and depressive or anxiety symptoms. Furthermore, given that the mean age of individuals with chronic diseases recruited in studies was 60 years old, these elders would consider Chinese culture as his or her Mother culture and tend to internalize cultural values (Fung, 2013) and might be inclined to Taoistic coping comparing with young people who grow in a multicultural society. Therefore, these elders were supposed to accept easily to apply Taoistic doctrines like self-transcendence, inaction, and the Law of Nature in their daily lives. The possibility that CTCT is less efficacious in younger samples is required to be proved in the further studies by recruiting younger adults with chronic diseases (e.g., chronic pain, obesity, and chronic hepatitis). Our results indicate that CTCT is an efficacious treatment for subjects with non-clinical symptoms of depression and can work together with pharmacological treatment to reduce depressive symptoms in subjects with MDD. However, no effect of CTCT on anxiety was found. Given the small number of studies focusing on anxiety disorders, the results may be biased and should be considered exploratory. Therefore, we could not make a conclusion on the efficacy of this therapy for anxiety disorders.

In addition, it should be noted that, at present, there is no a standard treatment session for CTCT. Most of included studies carried out therapy twice a week (for 4 weeks) or once a week (for 6 weeks or 8 weeks). Only two studies arranged CTCT monthly and continued 6 months or 1 year. Thus, we considered treatment session <8 weeks as a short-term therapy, otherwise it was a long-term therapy in our analysis. The preliminary evidence of the current study indicates that long-term CTCT intervention introduces a larger effect than short-term therapy, and its short-term efficacy seems to be less conspicuous than long-term efficacy. The possible reason was that subjects could reinforce what they learned in a longer period of time by a long-term CTCT training and thus handle Taoism skillfully in daily life. Similarly, if subjects could not review what they learned regularly, the outcome of more than 12 months follow-up work would not be desirable. However, long-term and longitudinal trials were limited, and the actual short-term efficacy and long-term efficacy of CTCT remain unclear. Further research that examines the efficacy of CTCT with different treatment sessions and treatment modalities (individual or group format) to optimize clinical practice is warranted.

Several overlooked methodological limitations of included studies should be acknowledged. First, publication bias is detected in several subgroup analyses. There are two possible reasons. First, both the number of included studies and the sample size of each study are small. If studies with small sample size are mainly in one direction (usually the direction of positive results), asymmetry will be observed in funnel plots and this may be indicative of publication bias (Light and Pillemer, 1984). Second, previous studies showed that negative results often failed to be reported compared with positive results (Song et al., 2009) and statistically positive findings were more likely published (Chan and Altman, 2005). We did not find any relevant unpublished studies when we searched in electronic databases and manually searched personal bibliographies or the reference lists of the included studies. Therefore, positive-results bias (a type of publication bias) may occur. Second, although the present results reveal that CTCT is effective in reducing depressive or anxiety symptoms for patients with chronic diseases, we could not overlook the possible selection bias as mentioned above. Future researches are recommended to investigate whether CTCT is effective for younger individuals (30–40 years old) with chronic diseases. Third, some included studies may have a low study quality due to the insufficient information on randomization and allocation concealment. In some cases it seems that study quality was difficult to assess given lack of details provided in the study. Future studies are encouraged to provide more details regarding the methods used to reduce risk of bias, including how random assignment was conducted, blinding of outcome assessment, etc. Fourth, arranging more than one therapist and an independent assessor is important for ensuring the competence of therapists and minimizing the possible effects of confounding factors. However, none of these studies described details of the implementation of treatment and therapist qualifications. Fifth, beyond the measurement of symptom reduction, quality of life and functioning evaluation should be emphasized by future studies. The two outcomes are essential to appraise this type of psychotherapy. Some economic evaluations, such as cost-effectiveness and cost-benefit, are also welcome to patients and clinical therapists to tailor personalized treatment regimens. In addition, the subjects were all from China, thereby possibly affecting the applicability of this therapy. We are unclear whether our results can be generalized to Chinese communities in other countries and in other Asian countries that share philosophies with China. Considering that different experiences of Chinese culture and knowledge may contribute to different interpretations of Taoism, a broader range of well-designed research is recommended. Finally, the mechanisms of action through which CTCT can improve psychological outcomes are unclear yet. Researchers conjectured that CTCT might benefit mental health through cognitive change, relaxation, acceptance, and self-transcendence (Wang et al., 2012). However, up to date, no study has examined how treatment effects occur during therapies through mediation analyses such as structural equation modeling (Kline, 2011) and causal-steps test (Baron and Kenny, 1986). It is important to conduct researches to establish mechanisms of CTCT in the future for the optimization and development of this therapy and the customization for individuals.

In conclusion, significantly positive effects of CTCT on depressive symptoms were revealed in our current preliminary meta-analysis, thereby suggesting that this therapy be used in the treatment of depressive symptoms across Chinese communities, including individuals with chronic physical diseases such as DM2, CHD, and stroke. Long-term treatment (more than 8 weeks) appears more effective than short-term treatment. However, the effectiveness of CTCT for anxiety symptoms remains debatable due to the very limited data. The results of our review show the need for additional well-designed studies, and further validation is invited. Our results may guide future studies in this field and help to construct a mature Chinese culture-oriented psychotherapy used in clinical practice.

## Author Contributions

WG, JC, and JZ designed the study. YD and LW searched for studies. YD analyzed these included data and wrote the first draft of the manuscript. All authors contributed to and have approved the final manuscript.

## Conflict of Interest

The authors declare that the research was conducted in the absence of any commercial or financial relationships that could be construed as a potential conflict of interest.
