# Supplementary material for: Chinese Taoist Cognitive Therapy for Symptoms of Depression and Anxiety in Adults in China: A Systematic Review and Meta-Analysis
Source: Front Psychol. 2020 Apr 23;11:769. doi: 10.3389/fpsyg.2020.00769 (PMC7192096; doi:10.3389/fpsyg.2020.00769)
Supplement: Supplementary file 1 [file Data_Sheet_1.docx]

Figure S1. Study flow chart.


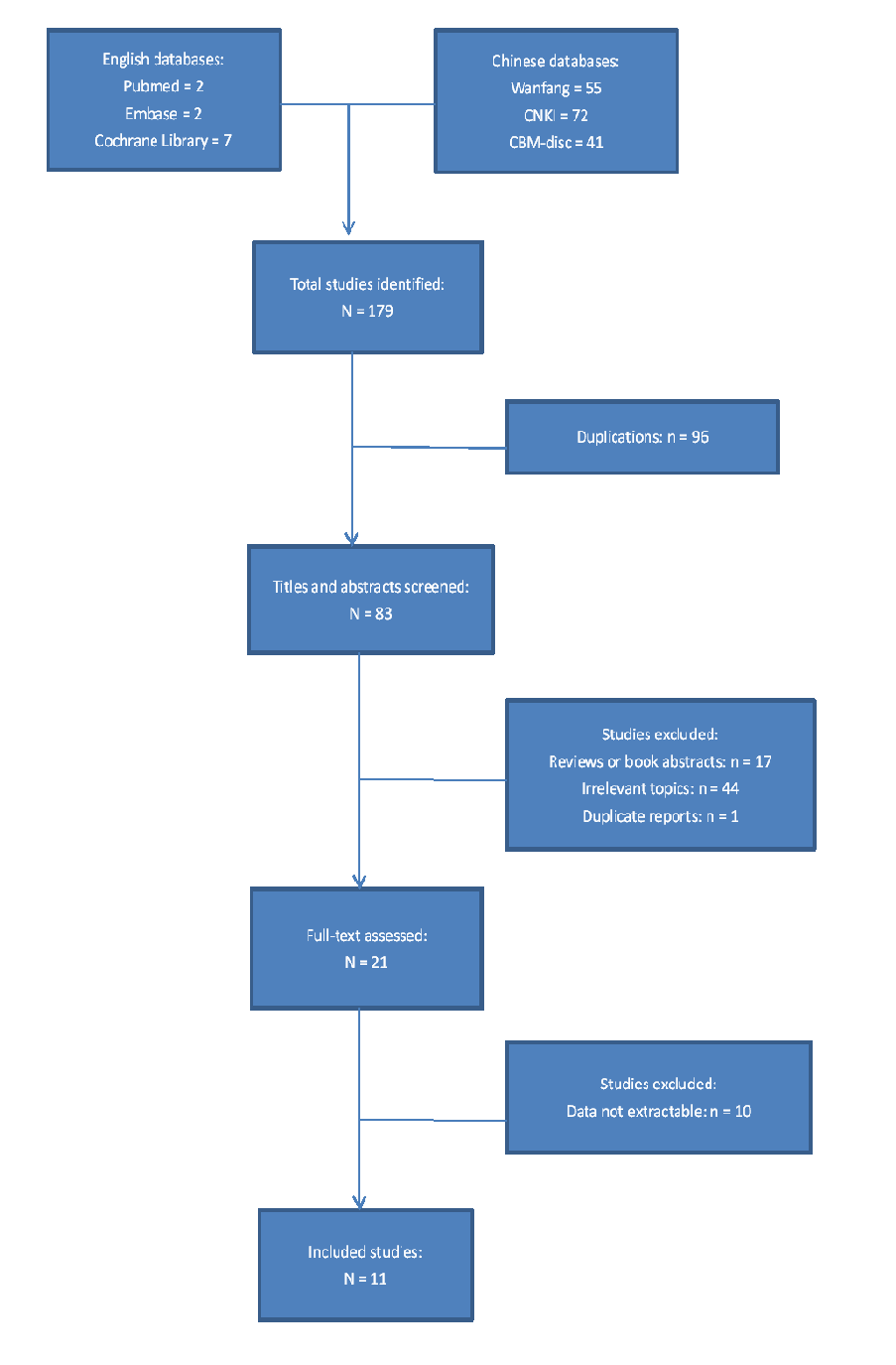


Abbreviations: CNKI = China National Knowledge Infrastructure; CBM-disc = Chinese Biology Medicine Database.

Figure S2. Forest plot (a) and Funnel plot (b) of 11 included studies.


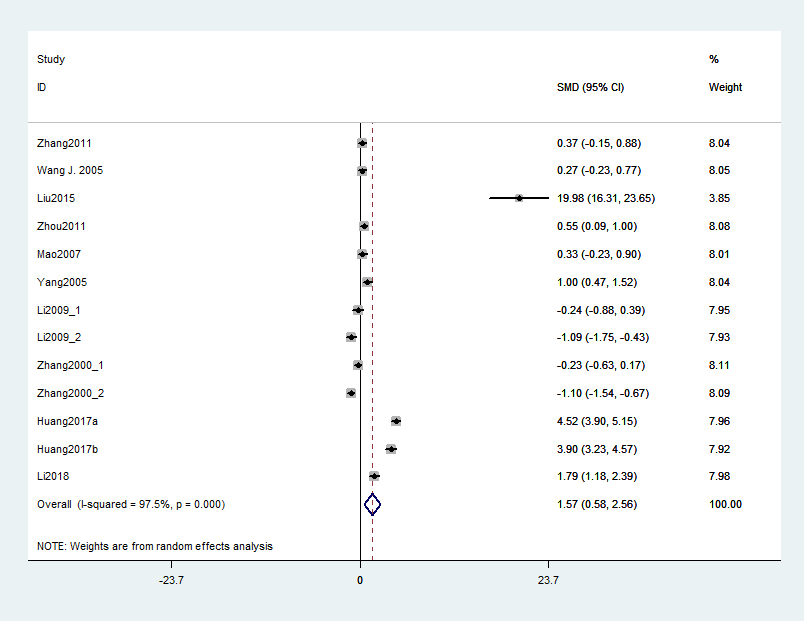


(a)


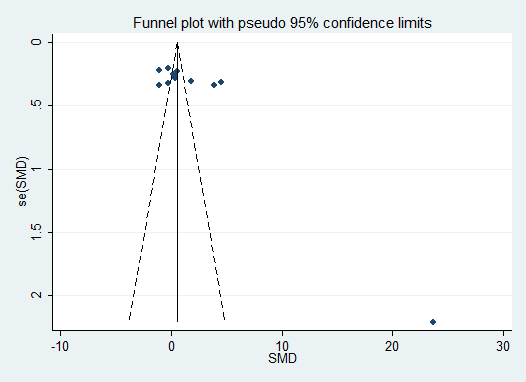


(b)

Abbreviation: SMD = standardized mean difference

Figure S3. Sensitivity analysis of 11 included studies.


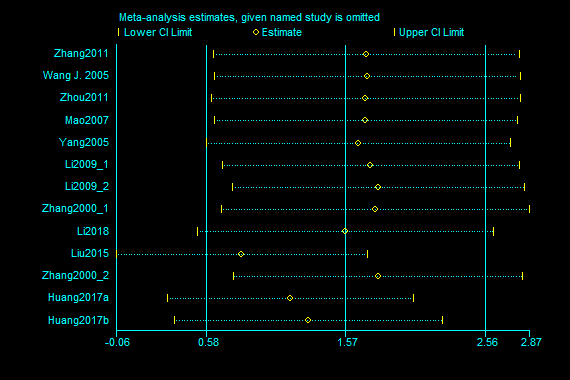


Figure S4. Funnel plots of subgroup analyses (a-h).


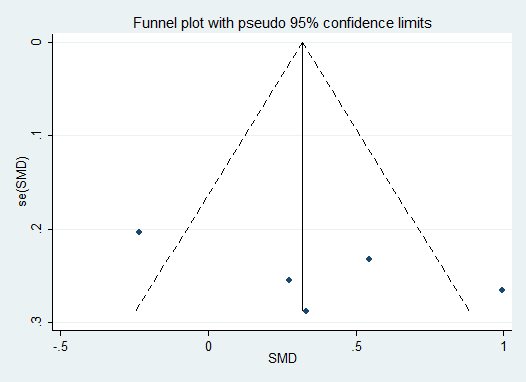


1. Funnel plot of CTCT + PMT vs. PMT alone group.


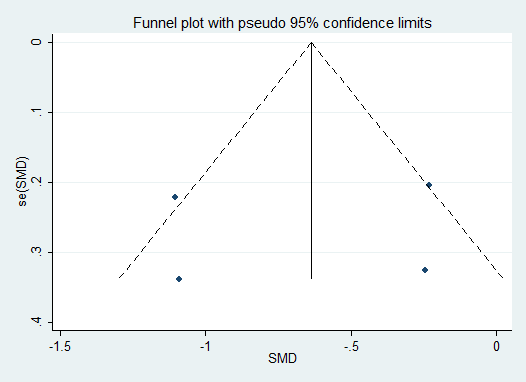


1. Funnel plot of anxiety disorders group.


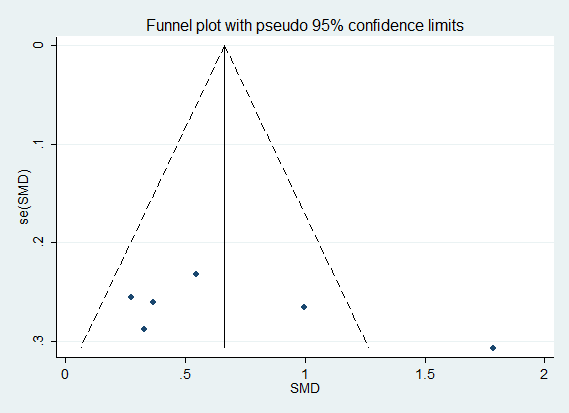


1. Funnel plot of Clinical/nonclinical depression group.


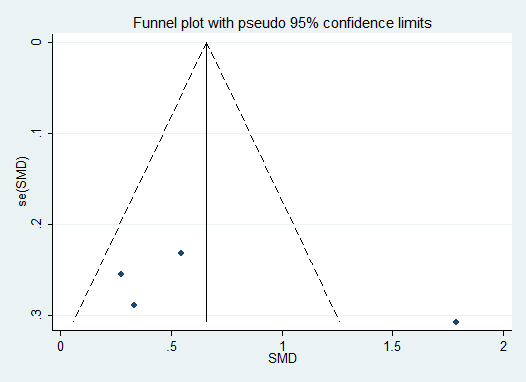


1. Funnel plot of chronic physical diseases group.


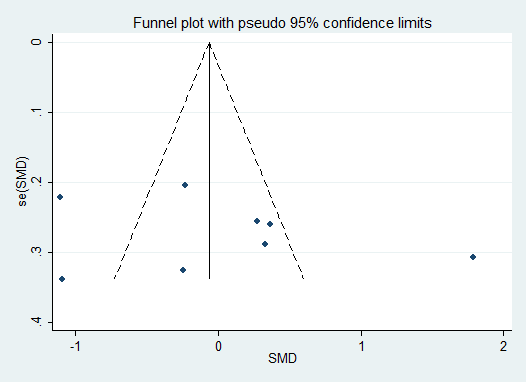


1. Funnel plot of short-term treatment ($<$8 weeks) group.


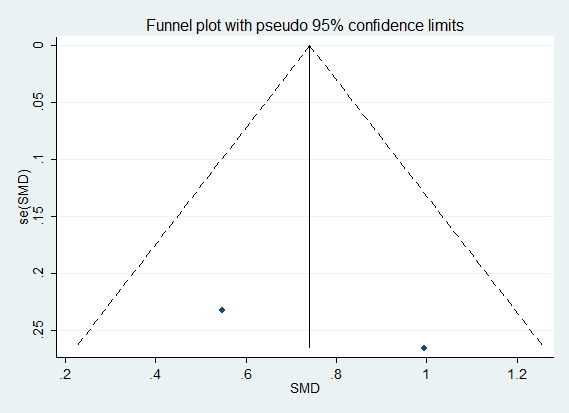


1. Funnel plot of long-term treatment ($\geq$8 weeks) group.


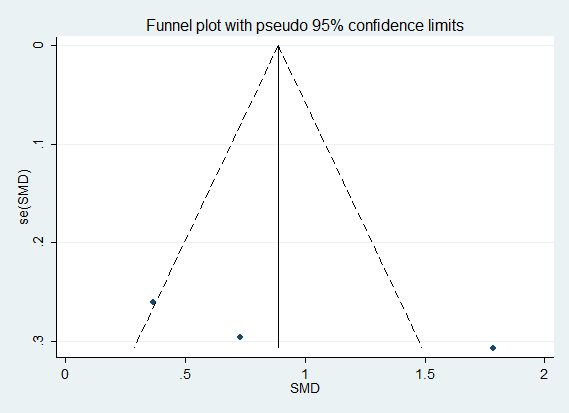


1. Funnel plot of short-term ($<$6 months) efficacy group.


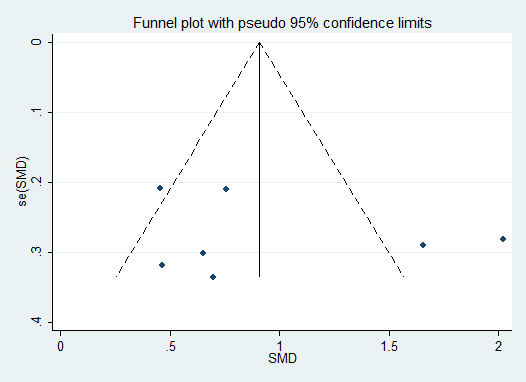


1. Funnel plot of long-term ($\geq$6 months) efficacy group.
